# Can Political Trust Weaken the Relationship between Perceived Environmental Threats and Perceived Nuclear Threats? Evidence from South Korea

**DOI:** 10.3390/ijerph18189816

**Published:** 2021-09-17

**Authors:** Jaeyoung Lim, Kuk-Kyoung Moon

**Affiliations:** 1 Department of Public Administration and Social Welfare, Chosun University, Gwangju 61452, Korea; jaeyounglim@yahoo.com; 2 Department of Public Administration, Inha University, Incheon 22212, Korea

**Keywords:** perceived environmental threats, perceived nuclear threats, political trust

## Abstract

As environmental movements rage, how to handle nuclear power plants has become a hotly contested issue globally. While concerns about nuclear power plants are warranted, nuclear power plants may play a crucial role in climate change discourse. In this context, this study examines the connections between individuals’ perceived environmental threats and their perceptions of the environmental threats posed by nuclear power plants (perceived nuclear threats). In particular, the study explores whether such connections are moderated by individuals’ level of political trust, such that political trust helps weaken perceptions of threats individuals may feel from nuclear power plants. Using the 2014 Korean General Social Survey and ordered probit, this study confirmed that individuals’ perceived environmental threats were positively associated with their perceived nuclear threats. Additionally, individuals with a high level of trust in the government can help alleviate the positive link between individuals’ perceived environmental threats and perceived nuclear threats.

## 1. Introduction

Countries around the world have experienced environmental calamities in recent years. Winter storms have hit regions rarely affected by such events, wildfires have become uncontrollable and severe floods have devastated regions around the world [1,2]. Experts predict climate refugees and farms without water [3,4]. Nuclear energy is considered to mitigate the impact of climate change [5]. However, several factors, such as the Windscale fire in England, Three Mile Island in the United States, Chernobyl in the former USSR and Fukushima in Japan, have increased concerns about nuclear energy’s dangers [6,7], increased the popularity of renewable energy sources [5] and led to the emergence of environmental movements against nuclear energy [5] that have combined into serious resistance against nuclear power plants. However, compared to fossil energy sources, nuclear energy remains vital to mitigate climate change’s harmful effects [5,8]. Citizens’ perceptions of environmental threats from nuclear power plants (perceived nuclear threats), therefore, take on important dimensions in the fight against environmental deterioration.

Within these contexts, this study explores what might influence individuals’ perceived nuclear threats by first focusing on the role of perceptions of environmental threats (perceived environmental threats). Defined as the perceived chance of harmful consequences caused by an environmental event [9], perceived environmental threats have been proven to be associated with individuals taking proactive actions and being willing to support pro-environmental behaviors [9,10,11,12]. Because individuals with a greater degree of perceived environmental threats are likely to be sensitive to potential threats from nuclear power plants, they might feel more vulnerable to such threats and consider them as much more severe than they actually are and they are likely to develop a greater degree of perceived nuclear threats.

Additionally, this study focuses on the role of political trust in explaining individuals’ perceived nuclear threats. Political trust has long been considered a vital heuristic that guides people to quickly judge governmental policies [13,14]. Because individuals do not possess sufficient time and resources to accurately assess given information regarding a government policy, political trust assists individuals’ decision making [13,14,15]. In this respect, when individuals place a high level of trust in the government, they likely believe that the government will properly handle situations surrounding nuclear power plants. Thus, individuals’ levels of political trust are expected to be negatively associated with individuals’ perceived nuclear threats.

In particular, this study explores whether the relationship between individuals’ perceived environmental threats and perceived nuclear threats is moderated by political trust. Because political trust functions heuristically, it helps individuals support governmental policies or programs, even though they find such policies or programs materially or ideologically risky [16,17,18]. After these heuristic acts, individuals feel less connection between their heightened sense of environmental threats and their perceived nuclear threats. Thus, we expect that individuals’ political trust moderates and helps weaken the link between individuals’ perceived environmental threats and perceived nuclear threats.

This study explores individuals’ perceptions in understanding their attitudes toward nuclear power plants. In particular, the study investigates the role political trust can play in mollifying individuals’ fear of environmental threats from nuclear power plants. Trust is an underappreciated yet vital component of our understanding of individuals’ attitudes toward energy policy preferences [19]. By uncovering aspects of political trust with respect to nuclear power preferences, this study offers policymakers a tool to consider when devising nuclear energy policies.

This study will proceed as follows. First, we examine the concepts of perceived environmental threats and perceived nuclear threats. Next, we explore the concept of political trust and the moderation role of political trust on the relationship between perceived environmental threats and perceived political threats. Then, using an empirical analysis, we test the hypotheses generated by theoretical explorations and we offer the results and their implications.

## 2. Materials and Methods

### 2.1. Literature Review and Hypotheses 

#### 2.1.1. Perceived Environmental Threats and Perceived Nuclear Threats

Both rich and poor countries alike have been ravaged by global warming and its dire consequences [3,4]. People globally have been subject to scorching heat, freezing and fires [3]; no country seems immune from the devastation effected by climate change [3,4]. Given these dire circumstances, energy source choice is increasingly important. While nuclear energy is not without disadvantages, it is considered to contribute to environmental sustainability and mitigate climate change [5]. Thus, the factors associated with individuals’ perceived nuclear threats are critical to the fight against climate change. 

This study focuses on individuals’ environmental threat perceptions in relation to their attitudes toward nuclear energy. Environmental threat perceptions can be defined as individuals’ perceptions of the likelihood of the adverse impacts that an environmental event may cause [9]. These perceptions are associated with a wide array of attitudes toward the environment [9,10,11,12].

In turn, the theoretical mechanisms linking environmental threat perceptions and environmental attitudes can be explained by protective motivation theory (PMT) [6,20,21,22], which resulted from an effort to understand the connections between individuals’ perceptions of threats and their behavioral choices [6,20,21,22]. According to this theory, individuals base their behaviors on their expectations of threats; they place expectancy values on perceived threats and adopt behaviors that deal with such threats [6,20,21,22].

PMT posits that individuals experience two cognitive processes—threat appraisal and coping appraisal—in perceiving threats [6,20,21,22,23]. Threat appraisal, in turn, comprises two components: threat vulnerability and threat severity [6,20,21,22,23]. Threat vulnerability refers to the likelihood of exposure that individuals perceive for a given threat; threat severity refers to the degree of individuals’ perceptions that such a threat would affect serious consequences [6,20,21,22,23]. In turn, individuals cope with threats through two mechanisms: response efficacy and self-efficacy [6,20,21,22,23]. Response efficacy refers to individuals’ perceptions of their behaviors in responding to threats; self-efficacy denotes individuals’ perceptions of their capacity to demonstrate such behaviors [6,20,21,22,23]. When individuals experience high degrees of threat appraisal and threat severity, they are highly likely to engage in coping mechanisms to address those threats [24,25].

PMT helps to understand the link between individuals’ environmental threat perceptions and their perceived nuclear threats. Individuals with a high degree of environmental threat perceptions may perceive a given threat as more threatening and severe. Such individuals may feel that they would be more threatened by and thus vulnerable to nuclear energy and the likelihood of adverse consequences of nuclear energy would be more severe for them. Thus, individuals with a high degree of environmental threat perception are likely to develop high perceived nuclear threats. While coping mechanism are not explored in this study, it can be expected that individuals with a greater degree of coping mechanisms—a high degree of response efficacy and self-efficacy—would exhibit pro-environmental behaviors [26] and willingness to avert or oppose nuclear energy [6].

Several studies have noted positive associations between environmental threat perceptions and pro-environmental behaviors [9,10,11,12]. For instance, individuals with a significant degree of environmental threat perceptions were associated with positive attitudes toward organic farming [11], environment-friendly products [10] and pro-environmental behaviors [9,27,28]. Others have identified positive relationships between individuals’ perceived environmental threats and nuclear energy preferences [5,6,29,30,31,32,33,34]. Environmental concern is negatively associated with supporting nuclear energy and positively associated with opposing nuclear energy [5]. Those alert to environmental threats would be motivated to oppose nuclear energy [6,29,30,31,32,33,34]. Thus, the following hypothesis is pertinent to an empirical investigation:

**Hypothesis** **1.** *Individuals’ perceived environmental threats are positively associated with their perceived nuclear threats.*

#### 2.1.2. Political Trust and Its Moderation on the Relationship between Perceived Environmental Threats and Perceived Nuclear Threats

Emerging in the 1970s, when citizens’ trust in political institutions began to plummet, political trust has since garnered broad scholarly attention [35,36]. Several events, including the Watergate Scandal, oil shocks and stagflation, engendered citizens’ disenchantment with mainstream political institutions [15]. In early scholarship on political trust, scholars examined what might influence political trust [35,36]. Since then, scholarly attention has shifted to political trust’s role, focusing on several outcomes of political trust [15,37,38]. Recently, scholars have explored political trust’s impact as a moderator on the relationships between explanatory and dependent variables [14,17]. The earlier focus on political trust as the dependent variable has changed to a focus on political trust’s functions as explanatory and moderating variables [18,39,40,41].

Political trust can be defined in three conceptually different approaches. First, political trust may reflect citizens’ feelings toward the government based on its performance [15]. For instance, citizens’ levels of political trust can be highly influenced by how the government handles major disasters, such as the COVID-19 pandemic. Second, political trust can be understood in terms of processes [42]. For instance, citizens may not like election outcomes, but they can be satisfied with the institutions and procedures governing such outcomes [42]. Third, political trust may reflect government commitment to integrity and transparency [38,40]. Citizens’ levels of political trust can surge if governments are corruption-free and are committed to transparency. For instance, if governments do not accurately update information on the confirmed number of COVID-19 cases and deaths, citizens may be confused about what to do, which may lead to rising political distrust. The definition of political trust therefore includes government performance and integrity and processes that buttress it and its functions.

Scholars in recent years have focused on political trust’s functions as explanatory and moderating variables because political trust functions as a heuristic device; a heuristic is a mental shortcut that enables individuals to judge a situation or policy effectively and quickly [18,39,40,41]. Because most individuals do not possess enough time, resources and capacity to assess given information accurately, they often rely on shortcuts [43]. For instance, people may purchase a particular TV more willingly on a friends’ recommendation. In this reasoning, political trust is a powerful heuristic through which individuals lend their support to governmental policies, even though they may not fully understand them [15]. Political trust is positively associated with a diverse array of government programs, ranging from racially targeted programs to social welfare programs [13,15]. Moreover, individuals with a high level of political trust are more inclined to support pro-environmental policies and exhibit pro-environmental behavior. Several studies have demonstrated relationships between political trust and nuclear power preferences [8,29,44,45,46,47,48,49,50,51]. For instance, a lack of political trust has been associated with aversion to nuclear power [8,29,44,45] and nuclear waste repositories [46], while political trust helped individuals accept nuclear power plants [47,48,49,50,51].

**Figure 1:**
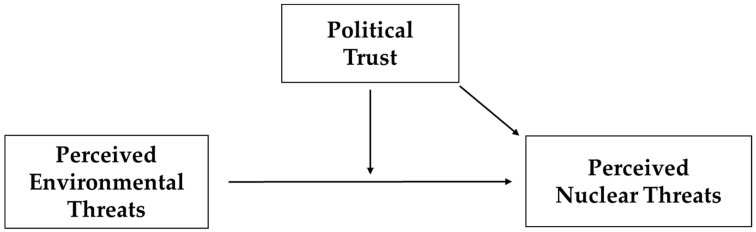
Conceptual Framework.

In particular, this study investigates whether political trust can play a role in moderating the relationship between individuals’ perceived environmental threats and perceived nuclear threats. The moderation mechanism can be explained as follows. Because political trust functions as a heuristic device [15,37], individuals are more likely to give credence to government policies handling nuclear power plants. Furthermore, ideological risk theory dictates that individuals who are ideologically or materially disposed to dislike a given policy may support it if they greatly trust the government [14,16,17,41]. Focusing on the heuristic power of political trust, scholars have noted that liberals may support tax cut policies and conservatives support social welfare programs tilted toward minorities if they have a greater level of trust in their political institutions [14,16,17,41]. Borrowing this reasoning and the heuristic role of political trust, it can be argued that individuals may be perceptually disinclined toward nuclear power plants due to their acute sense of environmental threats, but their risk perceptions of nuclear power plants can be significantly diminished if they place substantial confidence in their government. Thus, political trust can be expected to help weaken the positive relationship between individuals’ perceived environmental threats and perceived nuclear threats. Therefore, the following hypotheses can be generated for an empirical investigation. Figure 1 shows this study’s conceptual framework.

**Hypothesis** **2.** *Individuals’ levels of political trust are negatively associated with their perceived nuclear threats.*

**Hypothesis** **3.** *Political trust moderates the relationship between individuals’ perceived environmental threats and perceived nuclear threats, such that the relationship becomes weaker as the level of political trust increases.*

### 2.2. Data Collection

We used the 2014 Korean General Social Survey (KGSS) for our empirical analysis [52]. Administered by the Sungkyunkwan University Survey Research Center, the KGSS resembles the U.S. General Social Survey’s questions and format; core questions are repeated every survey, supplemented by topical questions for a given year [52]. Because the same respondents are not available for every survey, the KGSS is cross-sectional. From 2003 to 2014, the KGSS was implemented annually; after 2014, the survey has been administered biennially, except for 2020, when the survey was not run due to the COVID-19 pandemic [52]. We used the 2014 KGSS because it contained all the variables relevant to our study. This survey’s response rate was 55% and the sample size for our empirical model was 774. The survey employed area probability sampling, which takes population size into account. Finally, our model accounted for a weight.

### 2.3. Variables

#### 2.3.1. Perceived Nuclear Threats

The model’s dependent variable was individuals’ perceived nuclear threats, which comprised ordinal values, ranging from 1 to 5. It was based on one item: the respondents were asked how dangerous they thought nuclear power stations were to the environment. The variable is reverse-coded: 1 indicates “not dangerous at all” and 5 is “extremely dangerous.” The average of the dependent variable was 3.78, slightly above 3, thus suggesting that the respondents considered nuclear power stations to be environmentally threatening.

#### 2.3.2. Perceived Environmental Threats

This variable was a multi-item measure and a summed average of six items. Each item ranged from 1 to 5 and the variable ranged from 1.67 to 5. The respondents were asked their assessment of the danger posed to the environment by air pollution caused by cars, industry, pesticides and chemicals used in farming and by pollution of Korea’s rivers, lakes and streams, a rise in the world temperature caused by climate change and genetic modification of certain crops. The measure was highly reliable (Cronbach’s alpha = 0.826). The variable’s mean was 3.63, suggesting that the respondents had a high level of perceived environmental threats. We expected that individuals with a greater level of perceived environmental threats would take environmental threats from nuclear power plants more seriously.

#### 2.3.3. Political Trust

Political trust was a multi-item measure and a summed average of two items. The respondents were asked: “Thinking of the public service in South Korea, how committed is it to serve the people?” (ranging from 1 to 4) and “How widespread do you think corruption is in the public service in South Korea?” (ranging from 1 to 5). The two items were reverse-coded so that a lower value indicated distrust in the government and a higher value suggested trust. The variable ranged from 1 to 4.5 and its mean was 2.29, demonstrating that respondents distrusted the government more than trusted it. As political trust functions heuristically [15], we expected that individuals placing more trust in the government would be negatively associated with perceptions of nuclear power plants. More importantly, we contended that political trust would serve as the moderator of the link between individuals’ perceived environmental threats and perceived nuclear threats, as those with a greater level of political trust would support the government’s handling of nuclear power plants and this would weaken the perceptions individuals with a high level of perceived environmental threats might feel toward the danger of nuclear power plants to the environment. The measure was highly reliable (Cronbach’s alpha = 0.952).

#### 2.3.4. Control Variables

We controlled for several variables that may influence individuals’ perceptions of nuclear power plants. First, our model accounted for perceived local pollution. The variable was a multi-item measure and a summed average of perceptions of air, water and noise pollution in individuals’ communities. The measure was highly reliable (Cronbach’s alpha = 0.989). Individuals who are more cognizant of environmental pollution in their communities may have a higher perception of possible threats from nuclear power. some scholars have noted that individuals with greater awareness of local pollution are likely to have a high level of perception of environmental problems [53].

Second, we included political interest in our model. Political interest dictates that those who are acutely aware of contemporary issues may develop more empathy and understanding toward their resolution [54]. What to do with nuclear power plants has become a controversial agenda globally, including in South Korea, where conservatives and liberals have collided in recent years [55]. We expected that those with a greater level of political interest would understand the benefits of having nuclear energy and would be less likely to see nuclear energy as environmentally threatening. Knowledge of climate change is associated with attitudes toward supporting nuclear energy [5,56]. The variable was based on a single item: respondents were asked, “How interested would you say you are personally in politics?” A score of 4 indicated “very interested,” and a score of 1 indicated “not at all interested.”

Finally, the model accounted for the respondents’ demographics, namely gender, age, education and income. Women tend to be more sensitive to environmental threats than men [57], they are positively associated with opposing nuclear energy [5] and they are less likely to support the promotion of nuclear energy [56]. Gender was a dummy variable with female coded as 1 and male coded as 0. Women comprised 42% of the respondents. A meta study revealed that the relationship between age and environmental attitudes is inconclusive [58]. The respondents’ ages ranged from 18 to 83. Education was expected to have a positive impact on individuals’ perceptions of environmental threats; educated people are likely familiar with grave environmental issues facing the country and the surrounding communities [59], propelling them to be more perceptive of environmental threats presented by nuclear power plants. Education was expressed as levels: 0 indicated no formal schooling and 7 indicated doctoral graduate education. As for education, those who earn more may see environmental threats more acutely and nuclear power plants as an environmental menace. Thus, high-income earners may be positively associated with perceived nuclear threats. Income was also expressed as a level: 0 indicated no monthly income and 21 was a monthly income of more than KRW 10 million (roughly equivalent to USD 10,000 when USD 1 = KRW 1000). Table 1 describes the descriptive statistics of the variables included in the model.

### 2.4. Measurement Validity

We performed various statistical tests to ensure the validity of the measures used in our model. First, we performed Harman’s single factor test to detect whether our model suffered from common method variance. Because we relied on a single dataset that included all the variables in our model, common method variance could seriously threaten the model’s validity. However, the most dominant factor explained only 32.46% of the covariance in the model, indicating that the common method variance was not large enough to threaten the model’s validity. Second, the respondents were assured that their responses were anonymous, ensuring that they would not be pressured to produce desirable answers, which can result in the presence of common method variance [60]. Third, we performed confirmatory factor analysis to determine whether our model fit the data well. Our three-factor analysis—since the number of multi-item measures was three—yielded the following indices: RMSEA = 0.077, CFI = 0.929 and TLI = 0.904. These indices exceeded generally accepted thresholds [61], showing that our three-factor model fit the data reasonably well.

## 3. Results

We relied on an ordered probit for the statistical analysis of the model, since the dependent variable consisted of ordinal values. For our analysis, we relied on Stata 14 as a statistical program. The analysis also accounted for a weight reflective of population size and employed robust standard errors. The results are displayed in Table 2. The analysis proceeded in two steps. Step 1 explored the direct relationship between individuals’ perceived environmental threats and their perceived nuclear threats. Step 2 centered on the joint effects of individuals’ levels of political trust and perceived environmental threats on their perceived nuclear threats.

The results confirmed hypotheses 1 and 3. In Step 1, individuals with a high level of perceived environmental threats were positively associated with their perceived nuclear threats. Individuals perceiving environmental threats acutely are more likely to develop a stronger level of perceived nuclear threats, as they perceive nuclear threats’ exposure to the environment and the environmental severity of such threats more strongly based on the PMT. In contrast, Hypothesis 2 was not confirmed. Individuals’ levels of political trust were not significantly associated with perceived nuclear threats. While political trust was expected to function as a heuristic that may make individuals less sensitive to perceived nuclear threats, the results did not show that political trust was significantly associated with perceived nuclear threats. This suggests that political trust may not be a strong force that reshapes individuals’ attitudes toward threats directly presented by nuclear power plants to the environment.

In particular, the study focused on the joint effects of individuals’ perceived environmental threats and their level of political trust and how they jointly affect individuals’ perceived nuclear threats. Political trust functions as a mental shortcut that can moderate how individuals with an acute sense of environmental threats might perceive environmental threats generated by nuclear power plants. When individuals possess a high level of trust in the government, they might support its policies, even though they might disagree with them [14,16,17,41]. If this ideological risk theory is extended further, we can reasonably conjecture that even if individuals might be perceptually threatened by the presence of nuclear power plants, political trust can mollify their sense of threat, thanks to its heuristic function. Highly trusting individuals may think that the government would adequately handle issues associated with nuclear power plants and conclude that the latter may be less menacing to the environment. Thus, political trust helps alleviate the anxiety individuals with a high level of perceived environmental threats may have toward environmental threats from nuclear power plants.

In terms of controls, three variables—political interest, female sex and income—were significant in our model. First, individuals with a keen interest in political affairs might be aware of the potentially disastrous consequences of nuclear power plants when they are not properly regulated, resulting in a stronger level of perceived nuclear threats. Being female is also positively associated with perceived nuclear threats. Women tend to be more sensitive to environmental threats than men and the results confirm that women, at least in these data, are more perceptive of potential threats from nuclear power plants than men. Income also formed a positive relationship with the dependent variable, but the magnitude of the coefficients was small.

**Figure 2:**
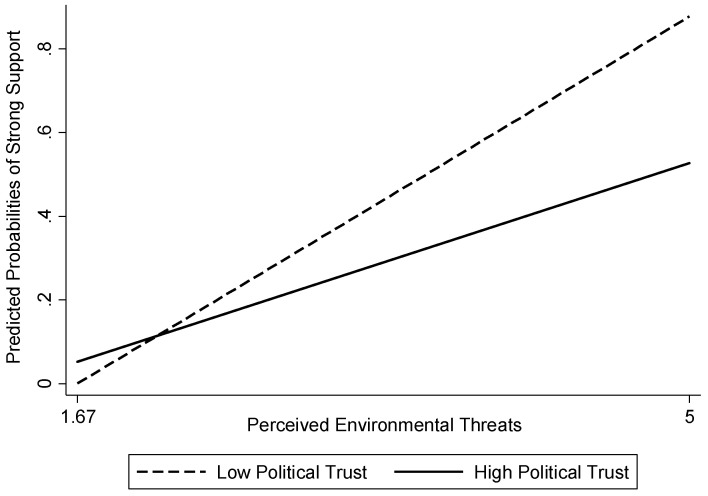
Predicted Probabilities of Strong Support for Perceived Nuclear Threats.

Figure 2 displays the joint effects of political trust and perceived environmental threats on perceived nuclear threats. The solid line refers to the relationship between perceived environmental threats and perceived nuclear threats when the level of political trust is high; the dashed line shows the relationship when the level of political trust is low.

In terms of the dashed lines, when the level of political trust is low, the relationship between perceived environmental threats and perceived nuclear threats is steep and strongly positive. The solid line shows that when the level of political trust is high, the relationship between perceived environmental threats and perceived nuclear threats is gradual rather than steep. In other words, as individuals’ perceptions of environmental threats increase, their perceptions of perceived nuclear threats increase, but the degree of that increase is much less steep when individuals place high trust in the government.

Individuals become less worried about threats posed by nuclear power plants to the environment when their trust level in the government is high compared to when that level is low. This visual presentation helps justify the need to examine the moderation of political trust in understanding the relationship between perceived environmental threats and perceived nuclear threats.

## 4. Discussion

The results indicate that policymakers should be concerned about improving citizens’ levels of political trust in the government. The level of political trust across countries, particularly developed ones, has significantly declined in recent decades [62,63]. Once this happens, it becomes significantly difficult for political actors to push for a given set of policies—perhaps even more so for policies related to nuclear power plants. Public discussions about nuclear power plants have become contentious globally [64]. What is missing in such discourses is a transparent appraisal of whether nuclear energy helps alleviate increasingly worrisome climate change around the world. For this, the recovery and enhancement of political trust are urgently needed.

Our study showed that political trust does not operate alone, but as a moderator of the link between perceived environmental threats and perceived nuclear threats. There are bound to be many individuals feeling threatened by the existence of nuclear power plants. For these individuals, political trust serves as a mollifying tool that helps them be less concerned about environmental threats from nuclear power plants. Policymakers, thus, need to work hard to earn citizens’ political trust. Since political trust is influenced by government performance [14,15], quick recovery of political trust will not be easy, as people’s perceptions of government performance can be long-term. It will require substantial systemic effort from policymakers to earn back citizens’ political trust. Still, the level of political trust can rise in national emergencies, such as pandemics [65]. For instance, the South Korean government superbly handled the pandemic in its early months and the government party benefited tremendously when it won a majority of seats in the April 2020 parliamentary election [66]. Its popularity has since plummeted due to a poor set of real estate regulations and scandals involving nominees for top government positions, resulting in a lower level of political trust [67]. The government is a lame duck, with few major policies expected to garner national support [67]. Because political trust is susceptible to changing circumstances, policymakers need to maintain a keen interest in policies and should be willing to push for them decisively when they enjoy a relatively high level of political trust [68].

## 5. Conclusions

Nuclear energy undeniably possesses the advantage over renewable energy of providing stable power [5,45] in many countries, while being a better alternative to fossil fuels for environmental sustainability. However, the severe consequences of nuclear power disasters, including the accident in Fukushima, have made many individuals feel threatened by the very presence of nuclear power plants [6,7]. Thus, alleviating individuals’ concerns about nuclear energy is becoming increasingly paramount in a global effort to mitigate the impact of climate change.

Facing these contexts, this study explored whether there is a link between individuals’ perceived environmental threats and perceived nuclear threats. More importantly, the study probed whether that link is moderated by individuals’ level of trust in the government so that individuals with a greater perceived level of political trust may find environmental threats posed by nuclear power plants less menacing.

The ordered probit results largely confirmed our hypotheses. First, we found that the more individuals perceive the environment as threatening, the more they perceive the environmental threats presented by nuclear power plants as threatening. Second, the study found that when contingent upon the level of political trust, individuals’ perceived environmental threats toward nuclear threats may be alleviated. Our findings reveal that political trust functions only as the moderator of the link between individuals’ perceived environmental threats and perceived nuclear threats. Although individuals sensitive to environmental risks possess a stronger level of perceived nuclear threats, their political trust helps alleviate such risks and makes them feel that environmental threats by nuclear power plants are less threatening.

Finally, we suggest several ideas for future studies. First, the dependent variable was based on a single item. Although some have argued that there is a high correlation between single item measures and multi-item measures, it would be ideal to use multi-item measures if such measures were available because of their desirable psychometric properties. Second, we relied on a single dataset, which may produce undesirable properties, such as common method variance and endogeneity. While we undertook Harman’s single factor test and a three-part confirmatory factor analysis and found that our model did not threaten measurement validity, using multiple datasets and collecting data in different periods would ensure a more rigorous study free of the potential presence of common method variance and endogeneity. Third, the main variables in our model were perceptual measures. Thus, the results do not guarantee actual outcomes. Future studies should employ actual outcome variables that would yield more valuable insights. Fourth, our study is based on cross-sectional data in one country. Naturally, causality between the explanatory and dependent variables is suspect at best. As such, the results of our study should be approached with caution. Finally, we focused on political trust as a moderating variable between perceived environmental threats and perceived nuclear threats, but it is also possible that the explanatory and dependent variables can be mediated by a factor other than political trust.

## Figures and Tables

**Table 1:** Descriptive Statistics.

Variables	*N*	Mean	SD	Min.	Max.
Perceived nuclear threats	774	3.78	0.94	1	5
Perceived environmental threats	774	3.63	0.62	1.67	5
Political trust	774	2.29	0.70	1	4.5
Perceived local pollution	774	2.45	0.63	1	4
Political interest	774	2.43	0.78	1	4
Age	774	44.99	13.20	18	83
Female	774	0.42	0.49	0	1
Income	774	6.45	4.22	0	21
Education	774	4.01	1.39	0	7

**Table 2:** Regression Results.

	Perceived Nuclear Threats			
	Step 1		Step 2	
	Coef.	(SE)	Coef.	(SE)
Perceived environmental threats	1.06	0.09 ***	1.71	0.28 ***
Political trust	0.05	0.07	1.02	0.44 **
Perceived environmental threats x Political trust			−0.27	0.12 **
Perceived local pollution	0.09	0.07	0.11	0.07
Political interest	0.16	0.06 ***	0.16	0.06 ***
Age	−0.00	0.00	−0.00	0.00
Female	0.35	0.09 ***	0.37	0.09 ***
Income	0.02	0.01 *	0.02	0.01 *
Education	−0.00	0.04	0.00	0.04
τ^1^	1.73	0.47	4.12	1.09
τ^2^	2.97	0.50	5.36	1.10
τ^3^	4.29	0.50	6.69	1.10
τ^4^	5.35	0.52	7.77	1.10
Log likelihood	−898.37		−893.54	
Wald test	162.73		234.39	
Number of cases	774			

* *p* < 0.1, ** *p* < 0.05, *** *p* < 0.01.

## Data Availability

The data used for this study are available at http://kgss.skku.edu/?page_id=39.
